# Family structure and birth order in relation to preschoolers’ social, emotional, and behavioral outcomes: a cross-sectional analysis of 19,987 children in western China

**DOI:** 10.3389/fpsyg.2026.1826209

**Published:** 2026-09-22

**Authors:** Jiali Du, Ke Sun, Hongli Sun, Huan Qiang

**Affiliations:** 1 College of Education Science, Yan'an University, UCSI University Malaysia, Yan'an, Shaanxi, China; 2 School of International Chinese Language Education, Yunnan University, Kunming, Yunnan, China; 3 Xi'an Key Laboratory of Children's Health and Diseases, Shaanxi Institute for Pediatric Diseases, Xi’an Children’s Hospital, Affiliated Children’s Hospital of Xi’an Jiaotong University, National Regional Children's Medical Center (Northwest), Xi’an, Shaanxi, China; 4 Department of Child Health Care, Xi'an Children's Hospital, Affiliated Children's Hospital of Xi'an Jiaotong University, National Regional Children's Medical Center (Northwest), Xi'an, Shaanxi, China

**Keywords:** a cross-sectional analysis, birth order, family structure, preschoolers, social, emotional, behavioral outcomes

## Abstract

**Introduction:**

Family structure and birth order are cross-sectionally associated with preschoolers’ social, emotional, and behavioral outcomes, yet their specific implications in diverse family contexts require further exploration.

**Methods:**

This cross-sectional study assessed social, emotional, and behavioral outcomes in 19,987 preschoolers (ages 3–6) across 189 kindergartens in western China using the Strengths and Difficulties Questionnaire (SDQ). Multivariate logistic and linear regression models were employed to evaluate associations, adjusting for demographic, socioeconomic, and behavioral covariates, with additional stratified and sensitivity analyses to test robustness.

**Results:**

A total of 19,987 preschoolers (51.6% boys) were included. Compared with only children, multi-child family status was associated with lower odds of prosocial behavior (OR = 0.90, 95% CI: 0.83–0.98, *p* = 0.016) and higher odds of externalizing problems (OR = 1.10, 95% CI: 1.00–1.21, *p* = 0.049) in fully adjusted models. No significant associations were found for total difficulties (OR = 0.97, *p* = 0.600) or internalizing problems (OR = 0.99, *p* = 0.880). Further analyses by birth order revealed that, compared with only children, second-born children showed modestly lower odds of total difficulties (OR = 0.65, 95% CI: 0.51–0.84, *p* = 0.001) and internalizing problems (OR = 0.75, 95% CI: 0.59–0.96, *p* = 0.019). Third-or-later-born children also exhibited a reduced risk of total difficulties (OR = 0.61, 95% CI: 0.38–0.98, *p* = 0.037). Conversely, first-born children were at increased risk of externalizing problems (OR = 1.30, 95% CI: 1.02–1.65, *p* = 0.034) and demonstrated lower prosocial behavior (OR = 0.82, 95% CI: 0.67–1.00, *p* = 0.048) compared to only children. Sensitivity analyses largely corroborated these findings, and no interactions remained significant after FDR correction (all q > 0.05).

**Conclusion:**

This cross-sectional study of 19,987 preschoolers found that multi-child families had lower prosocial behavior and higher externalizing risk than only children. First-born children showed higher externalizing risks and lower prosocial behavior, whereas second-born children showed the most favorable patterns, and third-or-later-born children showed lower odds of total difficulties.

## Background

The growing global prevalence of psychosocial challenges among preschool-aged children (3–6 years) presents a significant public health concern, impacting developmental trajectories, family dynamics, and social integration (Chen et al., 2024; Du Toit et al., 2025; Ma et al., 2024; Charach et al., 2020). During this critical neurodevelopmental period, environmental exposures are associated with these difficulties (Teicher et al., 2016; Qiao et al., 2024; Saputra et al., 2017). Understanding their potential correlates is essential for developing preventive interventions, particularly in socioeconomically disadvantaged regions like western China, where family environments necessitate targeted mental health support (Arbel et al., 2020). Compared with eastern coastal regions, western China exhibits substantially lower per capita household income, fewer licensed early childhood education institutions per capita, and higher rates of rural-to-urban parental migration, all of which may amplify the developmental significance of within-family resource distribution and sibling relationships (Fry and Blight, 2016; Huang et al., 2022).

The family environment shapes early psychological development, with parental factors such as anxiety, parenting style, and birth order influencing psychosocial outcomes (Coles and Cage, 2022). In western China, socioeconomic constraints, including lower household incomes and limited access to educational and mental health resources, and are associated with elevated risks of emotional and behavioral difficulties in preschoolers (Huang et al., 2022; Liu et al., 2021; Fry et al., 2012). The transition from China’s one-child policy to multi-child families has reshaped family dynamics, with only-child households offering concentrated resources but limited sibling-driven socialization, contrasting with multi-child families that foster emotional regulation through sibling interactions (Fu et al., 2024; Huang et al., 2022; Rodrigues et al., 2020). Birth order is hypothesized to be associated with psychosocial outcomes through differential socialization (Omer et al., 2022), with Adler’s theory suggesting firstborns experience psychological “dethronement” upon the arrival of siblings (Hsu et al., 2019; Greene and Clark, 1970). However, empirical support for these mechanisms is limited, particularly in western China’s unique socioeconomic and policy context. To date, large-scale studies examining birth-order effects on preschoolers’ psychosocial outcomes remain scarce in western China. While several nationwide surveys have compared only children with non-only children (Chen et al., 2024; Rao et al., 2024), these studies often lack region-specific analyses or disaggregated birth-order categories beyond the only-child/multi-child dichotomy. Consequently, whether Adlerian ‘dethronement’ effects and sibling socialization benefits observed in Western or East Asian coastal populations generalize to the distinct socioeconomic and cultural ecology of western China remains an open empirical question.

Despite these theoretical insights, prior studies on birth order and preschoolers’ mental health yielded inconsistent findings. Some research suggested firstborn and only children were more susceptible to psychosocial outcomes (Carballo et al., 2013), while others indicated only children faced fewer psychosocial difficulties due to concentrated parental attention (Rao et al., 2024). Other studies reported firstborn boys with ADHD showed heightened aggression in the presence of siblings, highlighting birth order’s role in psychosocial outcomes (Hsu et al., 2019). These inconsistencies arose due to small sample sizes, failure to distinguish only children as a reference group, and limited focus on non-Western family dynamics. In resource-constrained settings such as western China, family structure may be more strongly associated with child development through two primary pathways. First, resource dilution theory posits that limited economic and educational resources are subject to intensified competition among siblings, making birth-order effects more pronounced than in resource-abundant regions (Hotz et al., 2015; Downey, 1995). Second, the rapid policy transition from one-child to multi-child families in western China has occurred against a backdrop of lower baseline socioeconomic status, restricted access to early childhood education, and high rates of parental out-migration, potentially creating unique family dynamics where sibling interactions and grandparental co-parenting play compensatory roles (Liu et al., 2021). Thus, western China serves as a critical case for examining how macro-level policy shifts interact with micro-level family resource allocation to shape early childhood mental health.

This study employs a large-scale cross-sectional design to investigate how family structure, birth order, and demographic factors are associated with psychosocial outcomes in preschoolers aged 3–6 in western China. By comparing only-child and multi-child families, we aim to address gaps in prior research, which often overlooked only children as a sole reference group and failed to systematically compare these family types. Drawing on a sample of preschoolers in western China, where shifts from the one-child policy to multi-child households have reshaped family configurations, we examine whether birth order is associated with emotional regulation and social competence, and behavioral adjustment. We further explore whether interactions among these factors are associated with psychosocial outcomes.

## Methods

### Participants

We recruited 25,017 children aged 3–6 years from 189 kindergartens in Shaanxi Province using stratified sampling. Of these, 2,408 were excluded because the questionnaires were completed by non-parental guardians. An additional 1,058 children were excluded due to preterm birth (< 37 weeks gestation) or post-term birth (> 42 weeks gestation). We further excluded 436 children whose reported number of siblings was inconsistent with their birth order classification. Finally, 1,128 children were excluded due to biologically implausible values for parental or child age. After these sequential exclusions, a final sample of 19,987 children was included in the analysis. The study followed the Declaration of Helsinki, was approved by the Institutional Review Board at Xi’an Jiaotong University Children’s Hospital (Approval No. 20250225–21), all parents or legal guardians participating in the study signed written informed consent.

### Study measures

#### Birth order

Birth order was determined by the number and sequence of live births to the same biological parents. For the primary analysis, participants were divided into four categories: only children (no siblings), first-born, second-born, and third-or-later-born children (combining third-born, fourth-born, and beyond). However, we did not collect comprehensive information on the birth ranks of all siblings within the same household, which precludes more fine-grained analyses such as sibling-pair designs. A more granular five-category classification (distinguishing third-born and fourth-or-later-born children) was additionally used in sensitivity analyses.

#### Social, emotional, and behavioral outcomes

Strengths and Difficulties Questionnaire (SDQ) was distributed to each child’s father, mother, or other primary caregiver, with only one main guardian completing the survey per participant. The SDQ, with its 25 items, breaks down into five subcategories: emotional symptoms, conduct problems, hyperactivity/inattention, peer relationship problems, and prosocial behavior. Respondents rate each item on a three-point Likert scale—zero for “not true,” one for “somewhat true,” and two for “certainly true.” To derive the total difficulties score, the first four subscales are summed, with a score exceeding 14 suggesting the presence of clinically significant mental health issues, as per established Chinese normative standards (Wang et al., 2024; Du et al., 2008). Clinical thresholds specific to each subscale were established as follows: emotional symptoms were indicated by scores exceeding 3, conduct problems were denoted by scores greater than 2, hyperactivity/inattention was characterized by scores surpassing 6, peer problems were indicated by scores above 4, and prosocial behavior deficits was defined as scores below 6. Children were classified as being at risk (yes/no) for internalizing problems based on emotional symptoms, and for externalizing problems based on conduct problems or hyperactivity/inattention (Cheng et al., 2018; Haiwa Wang et al., 2024). The SDQ demonstrated strong psychometric properties in Chinese populations, as indicated by Cronbach’s α ranging between 0.72 and 0.82 across its different subscales. Additionally, the instrument displayed significant validity for children aged 3 to 17 years (Du et al., 2008; Goodman and Goodman, 2009).

### Covariates

Based on the risk factors for childhood social, emotional, and behavioral outcomes identified in previous literature, covariates were selected. Sociodemographic and behavioral data were collected through questionnaires. Parental gender was categorized as male or female. Parental age was recorded as a continuous variable in years. Child gender was classified as boys or girls, and child age was recorded as a continuous variable in years. Number of children in the family was categorized as one, two, or three or more. Education level was classified into three categories: junior high school or below, high school diploma or junior college, and undergraduate degree or above. Employment status was categorized as working, not working, or retired. Marital status was denoted as married/cohabiting or other (including single, divorced, separated, or widowed). Annual household income was segmented into three brackets: less than CNY 30,000, between CNY 30,000 and 60,000, and CNY 60,000 or more. Smoking status and alcohol intake status were each recorded as yes or no. Depressive symptoms were defined as a score of 16 or higher on the CES-D screening scale (yes/no). The parenting dysfunction score was dichotomized at the median into low and high groups. These covariates were selected based on established risk factors for childhood psychosocial difficulties identified in prior literature. Specifically, parental age, education, and employment status have been consistently associated with parenting quality and child mental health outcomes (O'Mahony et al., 2023). Household income and marital status reflect family economic stress and stability, which are linked to childhood internalizing and externalizing problems (Arbel et al., 2020). Additionally, parental smoking, alcohol intake parenting, dysfunction serve and depressive symptoms as indicators of family lifestyle and potential exposure to adverse home environments, both of which correlate with children’s behavioral regulation (Qiao et al., 2024).

### Statistical analysis

All questionnaire items were set as mandatory response fields, such that respondents could not submit the survey without completing all items; therefore, there were no missing data for any variable among eligible participants. Participants removed during the data-cleaning process (non-parental guardians, preterm or post-term birth, inconsistent sibling information, and implausible age values) were excluded entirely on the basis of predefined eligibility criteria rather than item-level missingness, and thus no imputation was required or performed. Baseline characteristics were compared across birth order groups (only children, first-born, second-born, and third-or-later-born) using analysis of variance for continuous variables and chi-square tests for categorical variables. Continuous variables were presented as means with standard deviations (SD), and categorical variables as frequencies with percentages.

Multivariate logistic regression models were fitted to estimate the associations between birth order and each outcome (total difficulties, internalizing problems, externalizing problems, and prosocial behavior), with odds ratios (ORs) and 95% confidence intervals (CIs) calculated. Two models were constructed: Model 1 was unadjusted; Model 2 was adjusted for 13 covariates, including parental age and gender, child age and gender, number of children, education level, employment status, marital status, annual family income, smoking status, alcohol intake status, depressive symptoms, and parenting dysfunction score.

To account for potential clustering of children within kindergartens, we additionally fitted multilevel logistic regression models with random intercepts at the kindergarten level. Intraclass correlation coefficients (ICCs) were computed from null models to quantify the proportion of variance attributable to between-kindergarten differences. Multicollinearity was assessed using variance inflation factor (VIF) diagnostics. Several sensitivity analyses were conducted to test the robustness of our findings: (1) linear regression models using continuous SDQ scores; (2) logistic regression models using a more granular five-category birth-order variable (distinguishing third-born from fourth-or-later-born children). Stratified analyses were performed to explore potential effect modification by demographic and socioeconomic factors, with interactions assessed by likelihood ratio tests comparing models with and without interaction terms. To account for multiple testing in the stratified analyses, the False Discovery Rate (FDR) Benjamini-Hochberg correction was applied. All statistical analyses were performed using R software (version 4.2.0). Two-sided *p*-values < 0.05 were considered statistically significant.

## Results

### Study participants

Our final analytical sample comprised 19,987 participants (10,313 boys, 51.6%; 9,674 girls, 48.4%), with 4,780 fathers (23.9%) and 15,207 mothers (76.1%) as primary respondents. The mean age was 4.82 ± 0.89 years for children and 34.74 ± 4.53 years for parents. As shown in Table 1, baseline demographic, socioeconomic, and behavioral characteristics differed significantly across birth order groups (only children, first-born, second-born, and third-or-later-born) for most variables, including parental age and gender, child age and gender, education level, employment status, marital status, annual family income, smoking status, alcohol intake, depressive symptoms, and parenting dysfunction score (all *p* < 0.05).

**Table 1:** Descriptive characteristics of participants by child ranking group.

Variables	Total (*N* = 19,987)	Only children(children without siblings) (*N* = 5,662)	First-born children (*N* = 3,102)	Second-born children (*N* = 9,559)	Third-or-later-born children (*N* = 1,664)	*p*-value	Effect
Parental gender							
Male	4,780 (23.9%)	1,526 (27%)	782 (25.2%)	2099 (22%)	373 (22.4%)	<0.001	0.052
Female	15,207 (76.1%)	4,136 (73%)	2,320 (74.8%)	7,460 (78%)	1,291 (77.6%)		
Parental age, years	34.74 ± 4.53	32.83 ± 3.79	32.43 ± 3.82	36.04 ± 4.31	38.06 ± 4.54	<0.001	0.175
Child gender							
Boys	10,313 (51.6%)	3,027 (53.5%)	1,350 (43.5%)	4,917 (51.4%)	1,019 (61.2%)	<0.001	0.087
Girls	9,674 (48.4%)	2,635 (46.5%)	1752 (56.5%)	4,642 (48.6%)	645 (38.8%)		
Child age, years	4.82 ± 0.89	4.66 ± 0.88	4.96 ± 0.87	4.86 ± 0.89	4.84 ± 0.88	<0.001	0.014
Number of children							
1	5,662 (28.3%)	5,662 (100%)	0 (0%)	0 (0%)	0 (0%)	<0.001	0.926
2	12,101 (60.5%)	0 (0%)	2,982 (96.1%)	9,119 (95.4%)	0 (0%)		
≥3	2,224 (11.1%)	0 (0%)	120 (3.9%)	440 (4.6%)	1,664 (100%)		
Education level							
≤ Junior high school	5,236 (26.2%)	892 (15.8%)	660 (21.3%)	2,741 (28.7%)	943 (56.7%)	<0.001	0.187
High school diploma and junior college	8,913 (44.6%)	2,568 (45.4%)	1,396 (45%)	4,392 (45.9%)	557 (33.5%)		
≥ Undergraduate degree	5,838 (29.2%)	2,202 (38.9%)	1,046 (33.7%)	2,426 (25.4%)	164 (9.9%)		
Employment status							
Working	14,663 (73.4%)	4,719 (83.3%)	2,218 (71.5%)	6,799 (71.1%)	927 (55.7%)	<0.001	0.122
Not working	4,876 (24.4%)	834 (14.7%)	817 (26.3%)	2,540 (26.6%)	685 (41.2%)		
Retirement	448 (2.2%)	109 (1.9%)	67 (2.2%)	220 (2.3%)	52 (3.1%)		
Marital status							
Married or cohabiting	19,458 (97.4%)	5,414 (95.6%)	3,060 (98.6%)	9,372 (98%)	1,612 (96.9%)	<0.001	0.073
Other	529 (2.6%)	248 (4.4%)	42 (1.4%)	187 (2%)	52 (3.1%)		
Annual family income, in thousands, CNY							
< 30	7,946 (39.8%)	1902 (33.6%)	1,192 (38.4%)	3,990 (41.7%)	862 (51.8%)	<0.001	0.081
≥ 30 to 60	8,130 (40.7%)	2,395 (42.3%)	1,294 (41.7%)	3,832 (40.1%)	609 (36.6%)		
≥ 60	3,911 (19.6%)	1,365 (24.1%)	616 (19.9%)	1737 (18.2%)	193 (11.6%)		
Smoking status							
No	17,016 (85.1%)	4,700 (83%)	2,643 (85.2%)	8,245 (86.3%)	1,428 (85.8%)	<0.001	0.039
Yes	2,971 (14.9%)	962 (17%)	459 (14.8%)	1,314 (13.7%)	236 (14.2%)		
Alcohol intake status							
No	16,729 (83.7%)	4,651 (82.1%)	2,589 (83.5%)	8,092 (84.7%)	1,397 (84%)	<0.001	0.029
Yes	3,258 (16.3%)	1,011 (17.9%)	513 (16.5%)	1,467 (15.3%)	267 (16%)		
Depressive symptoms							
No	16,922 (84.7%)	4,945 (87.3%)	2,651 (85.5%)	7,993 (83.6%)	1,333 (80.1%)	<0.001	0.058
Yes	3,065 (15.3%)	717 (12.7%)	451 (14.5%)	1,566 (16.4%)	331 (19.9%)		
Parenting dysfunction score							
Low	8,087 (40.5%)	2,600 (45.9%)	1,255 (40.5%)	3,607 (37.7%)	625 (37.6%)	<0.001	0.073
High	11,900 (59.5%)	3,062 (54.1%)	1847 (59.5%)	5,952 (62.3%)	1,039 (62.4%)		

Values are presented as mean ± SD for continuous variables and n (%) for categorical variables.

### Association between family structure, birth order and social, emotional, and behavioral outcomes

Table 2 shows that in fully adjusted models, multi-child family status was associated with lower odds of prosocial behavior (OR = 0.90, 95% CI: 0.83–0.98, *p* = 0.016) and higher odds of externalizing problems (OR = 1.10, 95% CI: 1.00–1.21, *p* = 0.049) compared with only children. No significant associations were found for total difficulties (OR = 0.97, *p* = 0.600) or internalizing problems (OR = 0.99, *p* = 0.880). Logistic regression analyses were performed to examine the associations between birth order and child behavioral outcomes (Table 3). In the fully adjusted model (Model 2), compared with only children, second-born children had significantly lower risks of total difficulties (OR = 0.65, 95% CI: 0.51–0.84, *p* = 0.001) and internalizing problems (OR = 0.75, 95% CI: 0.59–0.96, *p* = 0.019), whereas no significant association was observed for externalizing problems (OR = 0.98, 95% CI: 0.78–1.24, *p* = 0.881). Third-or-later-born children also showed a reduced risk of total difficulties (OR = 0.61, 95% CI: 0.38–0.98, *p* = 0.037). In contrast, first-born children exhibited a significantly higher risk of externalizing problems (OR = 1.30, 95% CI: 1.02–1.65, *p* = 0.034) and lower prosocial behavior (OR = 0.82, 95% CI: 0.67–1.00, *p* = 0.048) compared to only children. These findings suggest that birth order may play a differential role in child behavioral outcomes, with later-born children demonstrating some advantages over first-born and only children. Supplementary Table 1 shows that ICCs ranged from 0.0003 to 0.0018, indicating negligible clustering effects. Multicollinearity was assessed using variance inflation factor (VIF) diagnostics, with all VIF values < 5 (see Supplementary Table 2), indicating no substantial collinearity.

**Table 2:** Associations between family structure and social, emotional, and behavioral outcomes: results from logistic regression analyses.

Outcomes	Model 1	Model 2
Total difficulties		
Only children	Ref.	Ref.
Multi-child families	0.98 (0.90–1.06) 0.5857	0.97 (0.87–1.08) 0.5997
Internalizing problems		
Only children	Ref.	Ref.
Multi-child families	1.04 (0.97–1.13) 0.2863	0.99 (0.90–1.10) 0.8800
Externalizing problems		
Only children	Ref.	Ref.
Multi-child families	0.97 (0.91–1.05) 0.4577	1.10 (1.00–1.21) 0.0490
Prosocial behavior		
Only children	Ref.	Ref.
Multi-child families	0.95 (0.90–1.01) 0.1286	0.90 (0.83–0.98) 0.0160

Values are presented as OR (95%CI) *p*-value. Model 1 is unadjusted. Model 2 is adjusted for 13 covariates: parental gender, parental age, child gender, child age, rank of children, education level, employment status, marital status, annual family income, smoking status, alcohol intake status, depressive symptoms, and parenting dysfunction score.

**Table 3:** Associations between birth order and social, emotional, and behavioral outcomes: results from logistic regression analyses.

Outcomes	Model 1	Model 2
Total difficulties		
Only children(children without siblings)	Ref.	Ref.
First-born children	1.19 (1.06–1.32) 0.002	0.96 (0.74–1.25) 0.769
Second-born children	0.89 (0.82–0.97) 0.009	0.65 (0.51–0.84) 0.001
Third-or-later-born children	1.11 (0.96–1.27) 0.146	0.61 (0.38–0.98) 0.037
Internalizing problems		
Only children(children without siblings)	Ref.	Ref.
First-born children	1.29 (1.16–1.43) 0.000	1.08 (0.85–1.37) 0.556
Second-born children	0.93 (0.86–1.01) 0.105	0.75 (0.59–0.96) 0.019
Third-or-later-born children	1.26 (1.10–1.43) 0.001	0.85 (0.55–1.33) 0.470
Externalizing problems		
Only children(children without siblings)	Ref.	Ref.
First-born children	1.16 (1.05–1.28) 0.004	1.30 (1.02–1.65) 0.034
Second-born children	0.94 (0.87–1.01) 0.111	0.98 (0.78–1.24) 0.881
Third-or-later-born children	0.84 (0.74–0.96) 0.013	0.93 (0.60–1.46) 0.754
Prosocial behavior		
Only children(children without siblings)	Ref.	Ref.
First-born children	0.92 (0.84–1.00) 0.060	0.82 (0.67–1.00) 0.048
Second-born children	0.96 (0.90–1.03) 0.278	0.89 (0.73–1.09) 0.267
Third-or-later-born children	0.96 (0.86–1.07) 0.415	0.88 (0.61–1.28) 0.502

Values are presented as OR (95%CI) *p*-value. Model 1 is unadjusted. Model 2 is adjusted for 13 covariates: parental gender, parental age, child gender, child age, number of children, education level, employment status, marital status, annual family income, smoking status, alcohol intake status, depressive symptoms, and parenting dysfunction score.

### Sensitivity analyses

To assess the robustness of our findings, we conducted several sensitivity analyses. First, we employed a more granular five-category birth-order variable (Table 4). In the fully adjusted models, second-born children continued to show significantly lower odds of total difficulties (OR = 0.66, 95% CI: 0.51–0.84, *p* = 0.001) and internalizing problems (OR = 0.75, 95% CI: 0.59–0.95, *p* = 0.019). Additionally, third-born children exhibited significantly reduced odds of total difficulties (OR = 0.59, 95% CI: 0.37–0.94, *p* = 0.028). First-born children remained at elevated risk for externalizing problems (OR = 1.30, 95% CI: 1.02–1.65, *p* = 0.034) and showed reduced odds of prosocial behavior (OR = 0.82, 95% CI: 0.67–1.00, *p* = 0.048). However, the associations for first-born children with total difficulties and internalizing problems became non-significant in this disaggregated analysis, and no significant associations were detected for fourth-born children across any outcome.

**Table 4:** Sensitivity analysis: Logistic regression using 5-category child ranking variable.

Outcomes	Model 1	Model 2
Total difficulties		
Only children(children without siblings)	Ref.	Ref.
First-born children	1.19 (1.06–1.32) 0.002	0.96 (0.74–1.25) 0.77
Second-born children	0.89 (0.82–0.97) 0.009	0.66 (0.51–0.84) 0.001
Third-born children	1.06 (0.92–1.23) 0.412	0.59 (0.37–0.94) 0.028
Fourth-born children	1.64 (1.13–2.37) 0.01	0.81 (0.45–1.49) 0.505
Internalizing problems		
Only children (children without siblings)	Ref.	Ref.
First-born children	1.29 (1.16–1.43) < 0.001	1.08 (0.84–1.37) 0.556
Second-born children	0.93 (0.86–1.01) 0.105	0.75 (0.59–0.95) 0.019
Third-born children	1.2 (1.05–1.38) 0.008	0.82 (0.52–1.28) 0.382
Fourth-born children	1.91 (1.34–2.72) < 0.001	1.22 (0.69–2.14) 0.498
Externalizing problems		
Only children(children without siblings)	Ref.	Ref.
First-born children	1.16 (1.05–1.28) 0.004	1.3 (1.02–1.65) 0.034
Second-born children	0.94 (0.87–1.01) 0.111	0.98 (0.78–1.24) 0.88
Third-born children	0.84 (0.74–0.97) 0.016	0.94 (0.6–1.46) 0.77
Fourth-born children	0.85 (0.57–1.27) 0.429	0.89 (0.49–1.61) 0.693
Prosocial behavior		
Only children (children without siblings)	Ref.	Ref.
First-born children	0.92 (0.84–1) 0.06	0.82 (0.67–1) 0.048
Second-born children	0.96 (0.9–1.03) 0.278	0.89 (0.73–1.09) 0.267
Third-born children	0.96 (0.86–1.07) 0.466	0.88 (0.61–1.27) 0.497
Fourth-born children	0.92 (0.66–1.28) 0.63	0.9 (0.55–1.47) 0.674

Values are presented as OR (95%CI) *p*-value. Model 1 is unadjusted. Model 2 is adjusted for 13 covariates: parental gender, parental age, child gender, child age, number of children, education level, employment status, marital status, annual family income, smoking status, alcohol intake status, depressive symptoms, and parenting dysfunction score.

Second, we used linear regression models with continuous SDQ scores (Table 5). The results were largely consistent with the primary logistic regression analyses. Second-born children showed significantly lower total difficulties scores (β = −1.04, 95% CI: −1.48 to −0.59, *p* < 0.001), and first-born children showed lower prosocial behavior scores (β = −0.24, 95% CI: −0.44 to −0.03, *p* = 0.023) compared to only children. Third-or-later-born children also had significantly lower total difficulties scores in the continuous analysis (β = −1.28, 95% CI: −2.11 to −0.45, *p* = 0.002), consistent with the categorical primary analysis (OR = 0.61, *p* = 0.037). Overall, these sensitivity analyses confirmed the robustness of the core findings, though some estimates within the later-born subgroups were less precise due to smaller sample sizes.

**Table 5:** Sensitivity analysis: linear regression with continuous outcomes.

Outcomes	Model 1	Model 2
Total difficulties score		
Only children(children without siblings)	Ref.	Ref.
First-born children	0.5 (0.28–0.72) < 0.001	0.1 (−0.36–0.55) 0.681
Second-born children	−0.37 (−0.54–0.21) < 0.001	−1.04 (−1.48–0.59) < 0.001
Third-or-later-born children	0.16 (−0.12–0.43) 0.258	−1.28 (−2.11–0.45) 0.002
Prosocial behavior score		
Only children (children without siblings)	Ref.	Ref.
First-born children	−0.12 (−0.21–0.03) 0.011	−0.24 (−0.44−−0.03) 0.023
Second-born children	−0.03 (−0.1–0.04) 0.382	−0.08 (−0.28–0.11) 0.403
Third-or-later-born children	−0.03 (−0.14–0.09) 0.65	−0.06 (−0.44–0.31) 0.735

Values are presented as *β* (95%CI) *p*-value. Model 1 is unadjusted. Model 2 is adjusted for 13 covariates: parental gender, parental age, child gender, child age, number of children, education level, employment status, marital status, annual family income, smoking status, alcohol intake status, depressive symptoms, and parenting dysfunction score.

### Stratified and interaction analyses

Stratified analyses were performed to evaluate the stability of the associations across subgroups defined by parental gender, parental age, child gender, child age, education level, employment status, marital status, annual family income, smoking status, alcohol intake, depressive symptoms, parenting dysfunction score, and number of children. Although some interaction *p*-values were below 0.05, none remained significant after FDR correction (all q-values > 0.05; see Supplementary Table 3), suggesting that the observed associations did not differ substantially across the examined subgroups.

## Discussion

In this large cross-sectional study of 19,987 preschoolers from western China, multi-child family status was associated with lower odds of prosocial behavior and higher odds of externalizing problems compared with only children, while no significant associations were observed for total difficulties or internalizing problems. Birth-order analyses further revealed that, compared with only children, second-born children showed lower odds of total difficulties and internalizing problems, third-or-later-born children showed lower odds of total difficulties, whereas first-born children showed higher odds of externalizing problems and lower odds of prosocial behavior.

First-born children, compared to only children, showed higher odds of externalizing problems and lower odds of prosocial behavior in the fully adjusted models, although no significant associations were observed for total difficulties or internalizing problems. Findings from a Taiwanese study indicated that first-born children had a higher risk of total difficulties, internalizing problems, and externalizing problems, suggesting that this relationship may be linked to excessive parental protection in Taiwanese society (Wu et al., 2012). While the specific mechanisms may differ by cultural context, this finding aligns with our observation of higher odds among firstborns. In our western Chinese sample, this pattern is more plausibly attributed to the dual burden of resource dilution following the arrival of younger siblings and heightened parental expectations for firstborns to excel academically and behaviorally within resource-constrained households, consistent with the Confucian emphasis on firstborns as role models for younger siblings (Hotz et al., 2015; Kollhoff et al., 2026).

Second-born children, relative to only children, had lower odds of total difficulties and internalizing problems. Their odds of externalizing problems and prosocial behavior were not significantly different from those of only children. One hypothesis is that younger siblings interact more with older siblings, helping them improve their social skills (Lawson and Mace, 2010; Slaughter et al., 2015). Consistent with this, a meta-analysis demonstrated that theory of mind and peer popularity are positively associated with having older siblings (Slaughter et al., 2015). Third-or-later-born children, compared to only children, showed lower odds of total difficulties, but no significant associations were observed for internalizing problems, externalizing problems, or prosocial behavior. A Japanese study indicated that last-born children were less likely to exhibit mental health problems and more inclined to demonstrate prosocial behavior and resilience (Fukuya et al., 2021). This may be related to Confucian cultural norms, where older siblings model behavior and care for younger ones, potentially promoting adaptive behaviors in later-born children (Mack et al., 2025; Clementino et al., 2015). Although resource dilution theory would predict disadvantages for later-born children due to more dispersed parental attention, the compensatory role of older siblings’ direct caregiving and social scaffolding may offset these risks, particularly in the three-generational co-parenting context prevalent in western China where grandparental support further buffers resource constraints. Prior research indicated that younger siblings rely on older siblings, experiencing greater security, and that last-born children were more likely to receive increased emotional support from older siblings (Teti and Ablard, 1989; Gass et al., 2007). These potential mechanisms warrant investigation in future longitudinal studies with direct measures of sibling interaction quality and parental expectations.

Our stratified analyses revealed no statistically significant interactions between demographic factors and birth-order associations with children’s social, emotional, and behavioral outcomes. This finding indicates that the observed cross-sectional associations did not differ substantially across the examined subgroups, rather than demonstrating that the effects are universally stable or equivalent across all strata. This pattern may reflect that cultural factors, such as Confucian family dynamics, could outweigh demographic influences in this preschool cohort from western China (Mu et al., 2024). However, as with all null interaction results, this interpretation should be viewed with appropriate caution. Longitudinal studies are needed to explore whether demographic factors are associated with these outcomes over time or in diverse cultural contexts. It is also important to note that, given the large sample size, even modest differences may reach statistical significance. For example, the association between multi-child family status and externalizing problems was borderline significant with a small effect size, and the reduced odds of prosocial behavior among multi-child families should be interpreted as a small rather than clinically substantial difference. These findings should therefore be regarded as preliminary cross-sectional associations warranting replication rather than evidence of clinically meaningful effects.

The present cross-sectional findings must be interpreted within the specific socioeconomic and familial ecology of western China. Unlike the relatively affluent coastal provinces where prior birth-order research in China has concentrated, our sample region is characterized by lower household incomes, limited kindergarten-to-population ratios, and a high prevalence of left-behind children whose parents migrate to eastern cities for employment (Liu et al., 2009). In such contexts, sibling relationships may function as a critical emotional buffer when parental care is disrupted by labor migration. Moreover, grandparental involvement in childcare is markedly higher in western China than in urban nuclear families elsewhere (Song et al., 2025); this three-generational co-parenting structure may attenuate resource dilution effects for later-born children while simultaneously intensifying role expectations for firstborns as model siblings within the Confucian intergenerational hierarchy. However, as these mechanisms were not directly measured, this interpretation remains hypothetical and requires empirical testing in future studies.

## Strengths and limitations

This study has several limitations. First, the sample was recruited from a single province in western China, limiting generalizability. Second, multiple births were not excluded, and siblings born in close succession may share similar risk backgrounds, potentially biasing the estimates. Third, although we controlled for parenting dysfunction, depressive symptoms, and number of children, important contextual factors—such as sibling relationship quality, primary caregiver arrangements, and left-behind-child status—were not measured due to pragmatic constraints of the original survey design. Fourth, we could not account for region-specific confounders such as children’s left-behind status, grandparental care intensity, and variations in fertility policy implementation, which may distort the observed associations. Fifth, birth order and family size are inherently intertwined. Being first-born in a two-child family may differ substantially from being first-born in a larger family. Although we adjusted for number of children as a covariate, we did not collect information on each child’s specific birth rank within the family. Therefore, caution is warranted when interpreting the independent effects of birth order.

Despite these limitations, our study has notable strengths. The large sample enables reliable statistical tests. Using only children as the reference group clearly distinguishes developmental patterns associated with different birth orders. We adjusted for multiple confounders, conducted multilevel modeling to account for kindergarten-level clustering, applied FDR correction, and performed sensitivity analyses, all of which confirmed the robustness of our findings. These strengths provide valuable evidence for early childhood mental health practice and lay a foundation for future research.

## Conclusion

This cross-sectional study of 19,987 preschoolers found that family structure and birth order were cross-sectionally associated with early childhood mental health outcomes. Multi-child families showed lower odds of prosocial behavior and higher odds of externalizing problems compared with only children. First-born children exhibited higher odds of externalizing problems and lower odds of prosocial behavior, whereas second-born children showed the most favorable patterns, and third-or-later-born children showed lower odds of total difficulties. Given the cross-sectional design, causal inferences cannot be drawn. These findings may inform birth-order-sensitive family support strategies.

## Data Availability

The original contributions presented in the study are included in the article/Supplementary material, further inquiries can be directed to the corresponding author.

## References

[ref1] Arbel R. Margolin G. O'Connor S. G. Mason T. B. Leventhal A. M. Dunton G. F. (2020). The curvilinear effect of mothers' parenting stress and attunement on children's internalizing symptoms: a six wave study of mother-youth dyads across the transition to adolescence. Dev. Psychol. 56, 1316–1330. doi: 10.1037/dev0000932, 32324013

[ref2] Carballo J. J. García-Nieto R. Alvarez-García R. Caro-Cañizares I. López-Castromán J. . (2013). Sibship size, birth order, family structure and childhood mental disorders. Soc. Psychiatry Psychiatr. Epidemiol. 48, 1327–1333. doi: 10.1007/s00127-013-0661-7, 23407902

[ref3] Charach A. Mohammadzadeh F. Belanger S. A. Easson A. Lipman E. L. McLennan J. D. . (2020). Identification of preschool children with mental health problems in primary care: systematic review and meta-analysis. J. Can. Acad. Child Adolesc. Psychiatry 29, 76–105. PMC7213917 32405310

[ref4] Chen M. Y. He F. Rao W. W. Qi Y. Rao S. Y. Ho T. I. . (2024). The network structures of mental and behavioral problems among children and adolescents in China using propensity score matching: a comparison between one-child and multi-child families based on a nationwide survey. J. Affect. Disord. 360, 206–213. doi: 10.1016/j.jad.2024.05.121, 38797390

[ref5] Cheng S. Keyes K. M. Bitfoi A. Carta M. G. Koç C. Goelitz D. . (2018). Understanding parent-teacher agreement of the strengths and difficulties questionnaire (SDQ): comparison across seven European countries. Int. J. Methods Psychiatr. Res. 27. doi: 10.1002/mpr.1589, 29024371 PMC5937526

[ref6] Clementino M. A. Gomes M. C. Pinto-Sarmento T. C. Martins C. C. Granville-Garcia A. F. Paiva S. M. (2015). Perceived impact of dental pain on the quality of life of preschool children and their families. PLoS One 10:e0130602. doi: 10.1371/journal.pone.0130602, 26090927 PMC4474672

[ref7] Coles D. C. Cage J. (2022). Mothers and their children: an exploration of the relationship between maternal mental health and child well-being. Matern. Child Health J. 26, 1015–1021. doi: 10.1007/s10995-022-03400-x, 35303222

[ref8] Downey D. B. (1995). When bigger is not better: family size, parental resources, and children's educational performance. Am. Sociol. Rev. 60, 746–761. doi: 10.2307/2096320

[ref9] Du Y. Kou J. Coghill D. (2008). The validity, reliability and normative scores of the parent, teacher and self report versions of the strengths and difficulties questionnaire in China. Child Adolesc. Psychiatry Ment. Health 2:8. doi: 10.1186/1753-2000-2-8, 18445259 PMC2409296

[ref10] Du Toit S. Tomlinson M. Laurenzi C. A. Gordon S. Hartmann L. Abrahams N. . (2025). Psychosocial interventions for preventing mental health conditions in adolescents with emotional problems: a Meta-analysis. J. Adolesc. Health 76, 187–209. doi: 10.1016/j.jadohealth.2024.09.030, 39556075

[ref11] Fry D. Blight S. (2016). How prevention of violence in childhood builds healthier economies and smarter children in the Asia and Pacific region. BMJ Glob. Health 1, i3–i11. doi: 10.1136/bmjgh-2016-000188, 28588988 PMC5418648

[ref12] Fry D. McCoy A. Swales D. (2012). The consequences of maltreatment on children's lives: a systematic review of data from the East Asia and Pacific region. Trauma Violence Abuse 13, 209–233. doi: 10.1177/1524838012455873, 22899705

[ref13] Fu Z. W. Wang K. L. Du N. Y. Li Y. J. Duan J. J. Zhao S. X. (2024). Comparative study on emotional behavior and parental job stress of only-child and non-only-child preschool children. World J. Clin. Cases 12, 4642–4651. doi: 10.12998/wjcc.v12.i21.4642, 39070806 PMC11235493

[ref14] Fukuya Y. Fujiwara T. Isumi A. Doi S. Ochi M. (2021). Association of Birth Order with Mental Health Problems, self-esteem, resilience, and happiness among children: results from A-CHILD study. Front. Psych. 12:638088. doi: 10.3389/fpsyt.2021.638088, 33935832 PMC8079750

[ref15] Gass K. Jenkins J. Dunn J. (2007). Are sibling relationships protective? A longitudinal study. J. Child Psychol. Psychiatry 48, 167–175. doi: 10.1111/j.1469-7610.2006.01699.x, 17300555

[ref16] Goodman A. Goodman R. (2009). Strengths and difficulties questionnaire as a dimensional measure of child mental health. J. Am. Acad. Child Adolesc. Psychiatry 48, 400–403. doi: 10.1097/CHI.0b013e3181985068, 19242383

[ref17] Greene R. L. Clark J. R. (1970). Adler's theory of birth order. Psychol. Rep. 26, 387–390. doi: 10.2466/pr0.1970.26.2.387, 4924444

[ref18] Haiwa Wang B. Zhao J. Yu Z. Pan H. Saishuang Wu B. Qi Zhu B. . (2024). Types of on-screen content and mental health in kindergarten children. JAMA Pediatr. 178:8. 10.1001/jamapediatrics.2023.5220 PMC10696513 38048076

[ref19] Hotz V. J. Pantano J. C. Ackerberg D. A. Black S. Boustan L. P. Buchinsky M. . (2015). Strategic parenting, birth order, and school performance. J. Popul. Econ. 28, 911–936. doi: 10.1007/s00148-015-0542-3, 26366045 PMC4565797

[ref20] Hsu Y. C. Chen C. T. Yang H. J. Chou P. (2019). Family structure, birth order, and aggressive behaviors among school-aged boys with attention deficit hyperactivity disorder (ADHD). Soc. Psychiatry Psychiatr. Epidemiol. 54, 661–670. doi: 10.1007/s00127-018-1624-9, 30535676

[ref21] Huang Y. Wang Y. Chen C. Gao Y. Kc A. Wang X. . (2022). Association between violent discipline at home and risk of illness and injury in children: findings from a cross-sectional study in rural Western China. J. Interpers. Violence 37:NP11413-NP11435. doi: 10.1177/0886260521991895, 33576282

[ref22] Huang W. Zhou Y. J. Zou H. Y. Yang X. Xu H. Li G. H. . (2022). Differences in non-suicidal self-injury behaviors between only-child and non-only-child adolescents with mood disorders: a cross-sectional study. Zhongguo Dang Dai Er Ke Za Zhi 24, 806–811. doi: 10.7499/j.issn.1008-8830.2201106, 35894197 PMC9336615

[ref23] Kollhoff E. M. Poustka L. Meyer T. (2026). Results from the Nationwide German KiGGS study in children and adolescents show that myopia is associated with being an only child. Ophthalmic Epidemiol. 33, 146–152. doi: 10.1080/09286586.2024.2434242, 39637406

[ref24] Lawson D. W. Mace R. (2010). Siblings and childhood mental health: evidence for a later-born advantage. Soc. Sci. Med. 70, 2061–2069. doi: 10.1016/j.socscimed.2010.03.009, 20381935

[ref25] Liu Z. Li X. Ge X. (2009). Left too early: the effects of age at separation from parents on Chinese rural children's symptoms of anxiety and depression. Am. J. Public Health 99, 2049–2054. doi: 10.2105/AJPH.2008.150474, 19762669 PMC2759782

[ref26] Liu X. Yang C. Yang Y. Huang X. Wang Y. Gao Y. . (2021). Maternal depressive symptoms and early childhood development: the role of mother-child interactions among mother-child dyads in rural areas of central and Western China. PeerJ 9:e11060. doi: 10.7717/peerj.11060, 33850652 PMC8018251

[ref27] Ma N. Ji X. Shi Y. Wang Q. Wu J. Cui X. . (2024). Adverse childhood experiences and mental health disorder in China: a nationwide study from CHARLS. J. Affect. Disord. 355, 22–30. doi: 10.1016/j.jad.2024.03.110, 38548193

[ref28] Mack E. Scherrer V. Preckel F. (2025). How smart is my child? The judgment accuracy of parents regarding their children's cognitive ability. Child Dev. 96, 122–140. doi: 10.1111/cdev.14156, 39169818 PMC11693832

[ref29] Mu W. Qiao K. Wang H. Wang Z. (2024). Impact of birth order on risk-taking: evidence from China. Int. J. Psychol. 59, 717–725. doi: 10.1002/ijop.13140, 38760980

[ref30] O'Mahony A. Stephens C. Livingston V. Dempsey E. Boylan G. Murray D. (2023). Postnatal maternal mental health and postnatal attachment. Rural Remote Health 23:8126. 10.22605/RRH8126 36802669

[ref31] Omer M. Posti J. P. Gissler M. Merikukka M. Bärnighausen T. Wilson M. L. (2022). Birth order and pediatric traumatic brain injury. Sci. Rep. 12:14451. doi: 10.1038/s41598-022-18742-3, 36002560 PMC9402548

[ref32] Qiao T. Wang L. Chang J. Huang X. Wang X. Ye P. (2024). The effect of paternal co-parenting on preschool children's problem Behaviors: the chain-mediating role of maternal parenting burnout and psychological aggression. Psychol. Res. Behav. Manag. 17, 187–200. doi: 10.2147/PRBM.S451878, 38250635 PMC10800087

[ref33] Qiao T. Wang X. Ye P. (2024). Maternal psychological aggression, problem Behaviors, and the mediating roles of psychological resilience and self-control in preschoolers. Psychol. Res. Behav. Manag. 17, 4383–4398. doi: 10.2147/PRBM.S497806, 39735857 PMC11673682

[ref34] Rao W. W. He F. Qi Y. Lok G. K. I. Jackson T. Zheng Y. . (2024). Mental health among school children and adolescents in China: a comparison of one-child and multiple-children families from a nationwide survey. Asian J. Psychiatr. 100:104130. doi: 10.1016/j.ajp.2024.104130, 39083955

[ref35] Rodrigues L. P. Lima R. F. Silva A. F. Clemente F. M. Camões M. Nikolaidis P. T. . (2020). Physical fitness and somatic characteristics of the only child. Front. Pediatr. 8:324. doi: 10.3389/fped.2020.00324, 32670998 PMC7329988

[ref36] Saputra F. Yunibhand J. Sukratul S. (2017). Relationship between personal, maternal, and familial factors with mental health problems in school-aged children in Aceh province, Indonesia. Asian J. Psychiatr. 25, 207–212. doi: 10.1016/j.ajp.2016.10.025, 28262152

[ref37] Slaughter V. Imuta K. Peterson C. C. Henry J. D. (2015). Meta-analysis of theory of mind and peer popularity in the preschool and early school years. Child Dev. 86, 1159–1174. doi: 10.1111/cdev.12372, 25874384

[ref38] Song Q. Yue A. Jiang Y. Cui M. Gao Q. Ma J. (2025). Grandmaternal caregiving and early child development in rural China: the mediating role of parenting environment. BMJ Paediatr. Open 9:e003434. doi: 10.1136/bmjpo-2025-003434, 41052813 PMC12506156

[ref39] Teicher M. H. Samson J. A. Anderson C. M. Ohashi K. (2016). The effects of childhood maltreatment on brain structure, function and connectivity. Nat. Rev. Neurosci. 17, 652–666. doi: 10.1038/nrn.2016.111, 27640984

[ref40] Teti D. M. Ablard K. E. (1989). Security of attachment and infant-sibling relationships: a laboratory study. Child Dev. 60:1519. doi: 10.2307/1130940, 2612257

[ref41] Wang H. Zhao J. Yu Z. Pan H. Wu S. Zhu Q. . (2024). Types of on-screen content and mental health in kindergarten children. JAMA Pediatr. 178, 125–132. doi: 10.1001/jamapediatrics.2023.5220, 38048076 PMC10696513

[ref42] Wu Y. T. Chen W. J. Hsieh W. S. Chen P. C. Liao H. F. Su Y. N. . (2012). Maternal-reported behavioral and emotional problems in Taiwanese preschool children. Res. Dev. Disabil. 33, 866–873. doi: 10.1016/j.ridd.2011.11.018, 22236631

