# Supplementary material for: Family structure and birth order in relation to preschoolers’ social, emotional, and behavioral outcomes: a cross-sectional analysis of 19,987 children in western China
Source: Front Psychol. 2026 Sep 22;17:1826209. doi: 10.3389/fpsyg.2026.1826209 (PMC13638573; doi:10.3389/fpsyg.2026.1826209)
Supplement: Supplementary file 1 [file Table_1.docx]

### Supplementary Table 1. Variance components and intraclass correlation coefficients (ICC) from null models with kindergarten as random intercept

| **Outcome** | **ICC** | **Percentage** | **Interpretation** |
| --- | --- | --- | --- |
| **Total difficulties** | 0.0010 | 0.10% | Minimal (< 0.2%) |
| **Internalizing problems** | 0.0003 | 0.03% | Minimal (< 0.2%) |
| **Externalizing problems** | 0.0018 | 0.18% | Minimal (< 0.2%) |
| **Prosocial behavior** | 0.0010 | 0.10% | Minimal (< 0.2%) |

### Supplementary Table 2. Multicollinearity diagnostics for the regression model of preschoolers’ social, emotional, and behavioral outcomes

| **Covariate** | **VIF** |
| --- | --- |
| **Parental gender** | 2.413 |
| **Parental age** | 1.176 |
| **Child age** | 1.032 |
| **Child gender** | 1.002 |
| **Number of children** | 1.205 |
| **Education level** | 1.316 |
| **Employment status** | 1.276 |
| **Annual family income** | 1.131 |
| **Marital status** | 1.027 |
| **Depressive symptoms** | 1.038 |
| **Parenting dysfunction score** | 1.031 |
| **Smoking status** | 2.342 |
| **Alcohol intake status** | 1.777 |

Note: VIF < 5 indicates no multicollinearity; VIF between 5 and 10 indicates moderate multicollinearity; VIF > 10 indicates severe multicollinearity.

Abbreviations: VIF, variance inflation factor.

### Supplementary Table 3. Stratified interaction analysis with FDR correction

| **Outcomes** | **Stratification variable** | **P-interaction** | **FDR-q** |
| --- | --- | --- | --- |
| **Total difficulties** | **Parental gender** | 0.6793 | 0.8820 |
| **Total difficulties** | **Parental age** | 0.3743 | 0.88 |
| **Total difficulties** | **Child age** | 0.2252 | 0.88 |
| **Total difficulties** | **Child gender** | 0.4328 | 0.88 |
| **Total difficulties** | **Education level** | 0.4127 | 0.88 |
| **Total difficulties** | **Employment status** | 0.8651 | 0.8821 |
| **Total difficulties** | **Marital status** | 0.7656 | 0.8821 |
| **Total difficulties** | **Annual family income** | 0.013 | 0.169 |
| **Total difficulties** | **Smoking status** | 0.779 | 0.8821 |
| **Total difficulties** | **Alcohol intake status** | 0.7286 | 0.8821 |
| **Total difficulties** | **Depressive symptoms** | 0.6812 | 0.8821 |
| **Total difficulties** | **Parenting dysfunction score** | 0.7978 | 0.8821 |
| **Total difficulties** | **Number of children** | 0.6999 | 0.8821 |
| **Internalizing problems** | **Parental gender** | 0.9916 | 0.9916 |
| **Internalizing problems** | **Parental age** | 0.7902 | 0.8821 |
| **Internalizing problems** | **Child age** | 0.6652 | 0.8821 |
| **Internalizing problems** | **Child gender** | 0.5113 | 0.8821 |
| **Internalizing problems** | **Education level** | 0.28 | 0.88 |
| **Internalizing problems** | **Employment status** | 0.6052 | 0.8821 |
| **Internalizing problems** | **Marital status** | 0.1374 | 0.8821 |
| **Internalizing problems** | **Annual family income** | 0.0097 | 0.169 |
| **Internalizing problems** | **Smoking status** | 0.7589 | 0.8821 |
| **Internalizing problems** | **Alcohol intake status** | 0.504 | 0.8821 |
| **Internalizing problems** | **Depressive symptoms** | 0.44 | 0.88 |
| **Internalizing problems** | **Parenting dysfunction score** | 0.7725 | 0.8821 |
| **Internalizing problems** | **Number of children** | 0.0107 | 0.169 |
| **Externalizing problems** | **Parental gender** | 0.1735 | 0.82019 |
| **Externalizing problems** | **Parental age** | 0.6032 | 0.8821 |
| **Externalizing problems** | **Child age** | 0.4329 | 0.88 |
| **Externalizing problems** | **Child gender** | 0.8244 | 0.8821 |
| **Externalizing problems** | **Education level** | 0.4338 | 0.88 |
| **Externalizing problems** | **Employment status** | 0.3132 | 0.88 |
| **Externalizing problems** | **Marital status** | 0.6783 | 0.8821 |
| **Externalizing problems** | **Annual family income** | 0.1957 | 0.8480 |
| **Externalizing problems** | **Smoking status** | 0.1685 | 0.8202 |
| **Externalizing problems** | **Alcohol intake status** | 0.1168 | 0.8202 |
| **Externalizing problems** | **Depressive symptoms** | 0.8327 | 0.8821 |
| **Externalizing problems** | **Parenting dysfunction score** | 0.4087 | 0.88 |
| **Externalizing problems** | **Number of children** | 0.7844 | 0.8821 |
| **Prosocial behavior** | **Parental gender** | 0.5279 | 0.8821 |
| **Prosocial behavior** | **Parental age** | 0.079 | 0.6847 |
| **Prosocial behavior** | **Child age** | 0.5543 | 0.8821 |
| **Prosocial behavior** | **Child gender** | 0.3995 | 0.88 |
| **Prosocial behavior** | **Education level** | 0.7026 | 0.8821 |
| **Prosocial behavior** | **Employment status** | 0.8593 | 0.8821 |
| **Prosocial behavior** | **Marital status** | 0.5968 | 0.8821 |
| **Prosocial behavior** | **Annual family income** | 0.4335 | 0.88 |
| **Prosocial behavior** | **Smoking status** | 0.3241 | 0.88 |
| **Prosocial behavior** | **Alcohol intake status** | 0.163 | 0.8202 |
| **Prosocial behavior** | **Depressive symptoms** | 0.2792 | 0.88 |
| **Prosocial behavior** | **Parenting dysfunction score** | 0.0019 | 0.0988 |
| **Prosocial behavior** | **Number of children** | 0.0636 | 0.6614 |

Note: P-interaction values are from likelihood ratio tests comparing models with and without interaction terms.

FDR-q: False discovery rate (Benjamini-Hochberg) corrected P-values.

Abbreviations: FDR, false discovery rate; BH, Benjamini-Hochberg.
